# Peripheral CD19^+^ B-cell counts and infusion intervals as a surrogate for long-term B-cell depleting therapy in multiple sclerosis and neuromyelitis optica/neuromyelitis optica spectrum disorders

**DOI:** 10.1007/s00415-018-9092-4

**Published:** 2018-10-30

**Authors:** Gisa Ellrichmann, Jan Bolz, Maren Peschke, Alexander Duscha, Kerstin Hellwig, De-Hyung Lee, Ralf A. Linker, Ralf Gold, Aiden Haghikia

**Affiliations:** 1 Department of Neurology, St. Josef-Hospital, Ruhr-University Bochum, Gudrunstrasse 56, 44791 Bochum, Germany; 2 Department of Neurology, Friedrich-Alexander-University Erlangen, 91054 Erlangen, Germany

**Keywords:** Multiple sclerosis, Neuromyelitis optica, Neuromyelitis optica spectrum disorders, Monoclonal anti-CD20 antibody, CD19^+^ B-cell counts

## Abstract

**Background:**

With ocrelizumab another drug is available for the treatment of multiple sclerosis (MS). Little is known on the long-term use of ocrelizumab on immune cell subsets, and no surrogate markers are available. Rituximab (RTX) has been in off-label use for the treatment of MS, neuromyelitis optica (NMO) and neuromyelitis optica spectrum disorder (NMOSD) for > 10 years.

**Objective:**

We evaluated the long-term depletion and repopulation rate of peripheral CD19^+^ B-cells as a potential surrogate for the clinical outcome, and whether it may serve for dosage and time-to-infusion decision making.

**Methods:**

We evaluated the CD19^+^ and CD4^+^/8^+^ T-cell counts in *n* = 153 patients treated with RTX (132 MS, 21 NMO/NMOSD). The dosages ranged from 250 to 2000 mg RTX. Depletion/repopulation rates of CD19^+^ B-cells as well as long-term total lymphocyte cell counts, were assessed and corroborated with EDSS, ARR (annualized relapse rate), MRI, and time to reinfusion.

**Results:**

CD19^+^ B-cells’ repopulation rate significantly varied depending on the dosage applied leading to individualized application intervals (mean 9.73 ± 0.528 months). Low/absent CD19^+^ B-cell counts were associated with reduced ARR, EDSS, and GD^+^-MRI-lesions. Long-term B-cell-depleting therapy led to a transiently skewed CD4^+^/8^+^ T-cell ratio due to reduced CD4^+^ T-cells and absolute lymphocyte counts, which recovered after the second cycle.

**Conclusion:**

Our data suggest that CD19^+^ B-cell repopulation latency may serve as surrogate marker for individualized treatment strategies in MS and NMO/NMOSD, which proved clinically equally effective in our cohort as evaluated by previous studies.

## Introduction

Although the T-cell (Th1 and Th17) mediated pathogenesis of MS is well established, B-cells and the humoral immune involvement are also increasingly recognized as drivers of the autoimmune disease and the concomitant neurodegeneration [18]. Hence, therapies either exclusively targeting B-cells, such as rituximab, ocrelizumab, and ofatumumab, or B-cells along with T-cells, such as alemtuzumab have proven to be effective in clinical trials [6].

The recent approval of the humanized antibody ocrelizumab depleting CD20^+^ B-cells, based on its efficacy shown in two phase III clinical trials in relapsing–remitting MS (RRMS; OPERA I and II studies) and one in primary progressive MS (PPMS; ORATORIO study), led to more than ten compounds (nine substance classes) for the treatment of MS [7, 8, 11]. However, the principle of B-cell depletion with its predecessor rituximab (RTX)—a chimeric monoclonal anti-CD20 antibody that binds to cell surface CD20 and induces antibody dependent cell-mediated cytotoxicity—has long been off-label use in MS and other autoimmune disorders. Several phase II studies and case series have previously shown its efficacy in MS [8, 9]. More recently, retrospective studies analyzing large Swedish MS cohorts receiving RTX treatment for a mean time of ~ 22 months provided further evidence for its beneficial therapeutic effect in RRMS and progressive MS [16, 17]. The rapid onset of its therapeutic effect, as shown in MRI measures after RTX initiation suggests that the mechanism by which RTX exerts its therapeutic effects is based not only on depleting B-cells as potential antibody producing cells, but also their involvement in T-cell activation [2]. In addition to MS, RTX has lately proven effective in NMOSD, another autoimmune demyelinating disease of the central nervous system that in contrast to MS is mediated by antibodies directed to the astrocytic aquaporin-4 [12].

Using ocrelizumab, the re-dosing is carried out at a fixed interval and dosage (600 mg every 6 months). Unlike ocrelizumab, there is no standard protocol for RTX infusions. At our centers, it is a current mode of clinical practice to follow B-cell counts as a measure of RTX reinfusion.

The aim of our study was to evaluate repopulation rate of peripheral CD19^+^ B-cells, as well as other lymphocyte subtypes as a potential surrogate marker for individual application intervals in patients with MS and NMO/NMOSD treated with RTX.

## Patients and methods

### Study population

The study was approved by the local ethics committees (reg-no 4493-12), and was intended to identify biological surrogate markers for the efficacy of drugs in MS and NMO/NMOSD. For analyses of CD56^+^ NK-cells and Th1-cells in healthy controls and RRMS patients, ethical permit was approved by the local ethics committees (reg-no 15-5351).

Patients who had received RTX and fulfilling either the McDonald criteria for MS or the criteria proposed by Wingerchuk et al. for NMO/NMOSD were included into the study [14, 19]. RTX was administrated when other options where exhausted or MRI indicated a B-cell pathology. The dosage and application interval was generally based on B-cell repopulation (2–5%), but did not follow a standardized protocol and differed on timing of patients’ clinic visits, underlying disease activity, side effects, patients’ conditions and physicians’ decision. That is the reason, why patients’ clinical visit intervals and applied RTX dose might vary in a single patient. In general, earlier (~ 10 years ago) the RTX dosages were higher and resembled protocols used by oncologists for the treatment of lymphoma patients. All RTX doses up to 1000 mg were applied in a single infusion, higher doses were given equally distributed within a 2-week interval. Beside this protocol, no double-dosing was administrated. For inclusion treatment should at least be once between April 2006 and September 2016. For baseline data, the EDSS and MRI before the first RTX infusion were used. Patients were treated as in- or out-patients in two German MS centers (Department of Neurology of the St. Josef-Hospital, Ruhr-University Bochum and the Department of Neurology, Friedrich-Alexander-University Erlangen). Anonymized patients’ data were used for stratification analyses, i.e. treatment response.

### Blood sampling and flow cytometry

Blood was taken at least twice in every patient in 3.5-ml EDTA tubes (Kabe, Germany) and a total of 893 blood tests were available. For absolute lymphocyte counts, samples were processed within 3 h and for flow cytometry (CD19^+^, CD4^+^, CD8^+^) at least within 24 h. B-cells were represented by CD19^+^ B-cells. Reagents used: eBioscience: BD Multitest™ Truecount CD3/CD16^+^CD56/CD45/CD19; reagent contains FITC-labeled CD3, clone SK7; PE-labeled CD16, clone B73.1, and PE-labeled CD56, clone NCAM 16.2 PerCP-labeled CD45, clone 2D1 (HLe-1); and APC-labeled CD19, clone SJ25C1.

For T-, NK-, B-, and Th1-cells, whole blood cells were stained by BD Multitest™ 6-Color TBNK Truecount (αCD3-FITC; αCD16-PE + αCD56-PE; αCD45-PerCP Cy5.5; αCD4-PE Cy7; αCD19-APC; αCD8-APC Cy7; BD) and afterwards analyzed in respect to absolute cell count numbers. Th1-cells were investigated after 4 h of restimulation with 50 ng/ml PMA and 1 µg/ml Ionomycin (both Sigma-Aldrich) and inhibition of vesicular transport by 1 µg/ml Monensin via the extracellular staining of whole blood cells with αCD4–FITC (RPA-T4; BD) and intracellular with α-IFNγ–APC (B27; BD) by usage of Foxp3/Transcription Factor Staining Buffer Set (eBioscience). For Treg analysis, cells were stained with CD4–FITC (RPA-T4, eBioscience) and PE Cy7 CD25–APC (BC96, eBioscience) extracellular and FoxP3–PE (236A/E7, eBioscience) intracellular by usage of Foxp3/Transcription Factor Staining Buffer Set (eBioscience) according to manufacturers’ protocol. For absolute cell numbers, cell counts of CD4^+^ IFNγ^+^-as well as CD4^+^ CD25^+^ FoxP3^+^ cells were used from FACS Diva v6 analysis. Lysis of erythrocytes in all samples was accomplished using FACS Lysing Solution (BD). For each sample, 5000 total events were recorded for analysis. All phenotyping experiments were performed on BD FACS Canto2 (BD Bioscience, Heidelberg) and analyzed by BD FACS DIVA v6 or BD FACS CANTO2/3 software.

### Stratification

The respective absolute long-term lymphocyte cell counts (cells/µl) were corroborated with disability progression (EDSS), MRI and ARR (annualized relapse rate). A relapse was defined as a worsening of clinical symptoms that lasted longer than 24 h and was not related to any infectious disease. We recorded the change in number of T1- and T2-weighed lesions as well as numbers of gadolinium enhancing (GD^+^) T1-lesions. EDSS, cerebral and spinal MRI compared to baseline were assessed before RTX therapy and after 12, 24 and 36 months.

### Statistics

Statistical analyses were performed using GraphPad Prism 6.0. Data are presented as a mean ± standard deviation (SD). For statistical analysis, group differences were evaluated using one-way analysis of variance (ANOVA) followed by Bonferroni’s post-hoc test (GraphPad Prism 6.0 software, San Diego, CA, USA). The probability levels of p* ≤ 0.05, p** ≤ 0.01, and p*** ≤ 0.001 were considered statistically significant for all statistical tests.

## Results

### Patients’ characteristics and treatment

A total of 153 patients treated with at least one course of B-cell depleting RTX therapy were included in our study (Table 1). Data of 21 NMO/NMOSD patients and 132 MS patients were analyzed. 72 of the MS patients were diagnosed as RRMS and 60 of them as secondary progressive MS (SPMS). Mean age of patients at disease onset was 33.58 ± 11.97 years and mean age at first RTX infusion was 41.69 ± 12.35 years. Mean follow-up was 26.28 ± 21.08 months. Mean RTX dose per treatment course was 717 mg ± 456 taking every infusion into account. If RTX infusion within a 2-week interval is regarded as one cycle, mean RTX dose is higher (828 mg ± 589) (Table 2). We did not observe differences in treatment response or dosing depending on previous therapy. As there would be too many subgroups resulting in small patient numbers per group, we decided to forego those subanalyses concerning pre-treatment.

**Table 1:** Patients’ characteristics

	*n* (%)	Mean ± SD
Number of patients	153 (100)	
Gender (female)	92 (62.4)	
Disease duration (years) at first RTX infusion		8.17 ± 8.02
Mean age (years) at disease onset		33.58 ± 11.97
Mean age (years) at first RTX infusion		41.69 ± 12.35
Follow-up (months)		26.28 ± 21.08
RRMS	72 (45.9)	
Gender (female)	50 (69.5)	
Disease duration (years) at first RTX infusion		6.38 ± 5.89
Mean age (years) at first RTX infusion		36.56 ± 9.49
Follow-up (months)		24.28 ± 19.52
SPMS	60 (38.2)	
Gender (female)	28 (46.6)	
Disease duration (years) at first RTX infusion		12.93 ± 9.71
Mean age (years) at first RTX infusion		45.93 ± 10.81
Follow-up (months)		28.02 ± 23.97
Patients with relapses in SPMS	26 (43.3)	
NMO/NMOSD	21 (13.3)	
Gender (female)	14 (66.7)	
Disease duration (years) at first RTX infusion		3.09 ± 4.72
Mean age (years) at first RTX infusion		44.24 ± 17.11
Follow-up (months)		30.81 ± 19.13

**Table 2:** Dosage and application intervals

	*n* = (%)	Application intervals (months)
All patients		
Number of RTX infusions		
1st course	153	
2nd course	130	9.7 ± 4.5
3rd course	93	9.8 ± 5.2
During whole follow-up	521	9.7 ± 4.72
Single RTX dosage (mg)		
250	98 (18.8)	6.4 ± 2.1
500	207 (39.7)	9.1 ± 4.4
1000	153 (29.4)	14.7 ± 6.7
> 1000	63 (12.1)	11.4 ± 2.0
RRMS		
Number of RTX infusions		
1st course	72	
2nd course	59	
3rd course	47	
During whole follow-up	269	
Single RTX dosage (mg)		
250	60 (22.3)	6.4 ± 2.6
500	110 (40.9)	8.7 ± 3.7
1000	62 (23.0)	14.3 ± 6.5
> 1000	37 (13.8)	11 ± 3.1
SPMS		
Number of RTX infusions		
1st course	60	
2nd course	54	
3rd course	29	
During whole follow-up	187	
Single RTX dosage (mg)		
250	38 (20.3)	7.2 ± 2.6
500	97 (51.9)	9.3 ± 3.4
1000	43 (23.0)	13 ± 7.4
> 1000	9 (4.8)	11 ± 9.3
NMO/NMOSD		
Number of RTX infusions		
1st course	21	
2nd course	17	
3rd course	17	
During whole follow-up	65	
Single RTX dosage (mg)		
1000	48 (73.8)	7.2 ± 3.5
> 1000	17 (26.2)	9.5 ± 3.7

Data are expressed as mean ± SD where appropriate

*n* number of patients, *NMO* neuromyelitis optica, *NMOSD* neuromyelitis optica spectrum disease, *RRMS* relapsing–remitting MS, *RTX* rituximab, *SPMS* secondary progressive MS

### Cell population

**Fig. 1:**
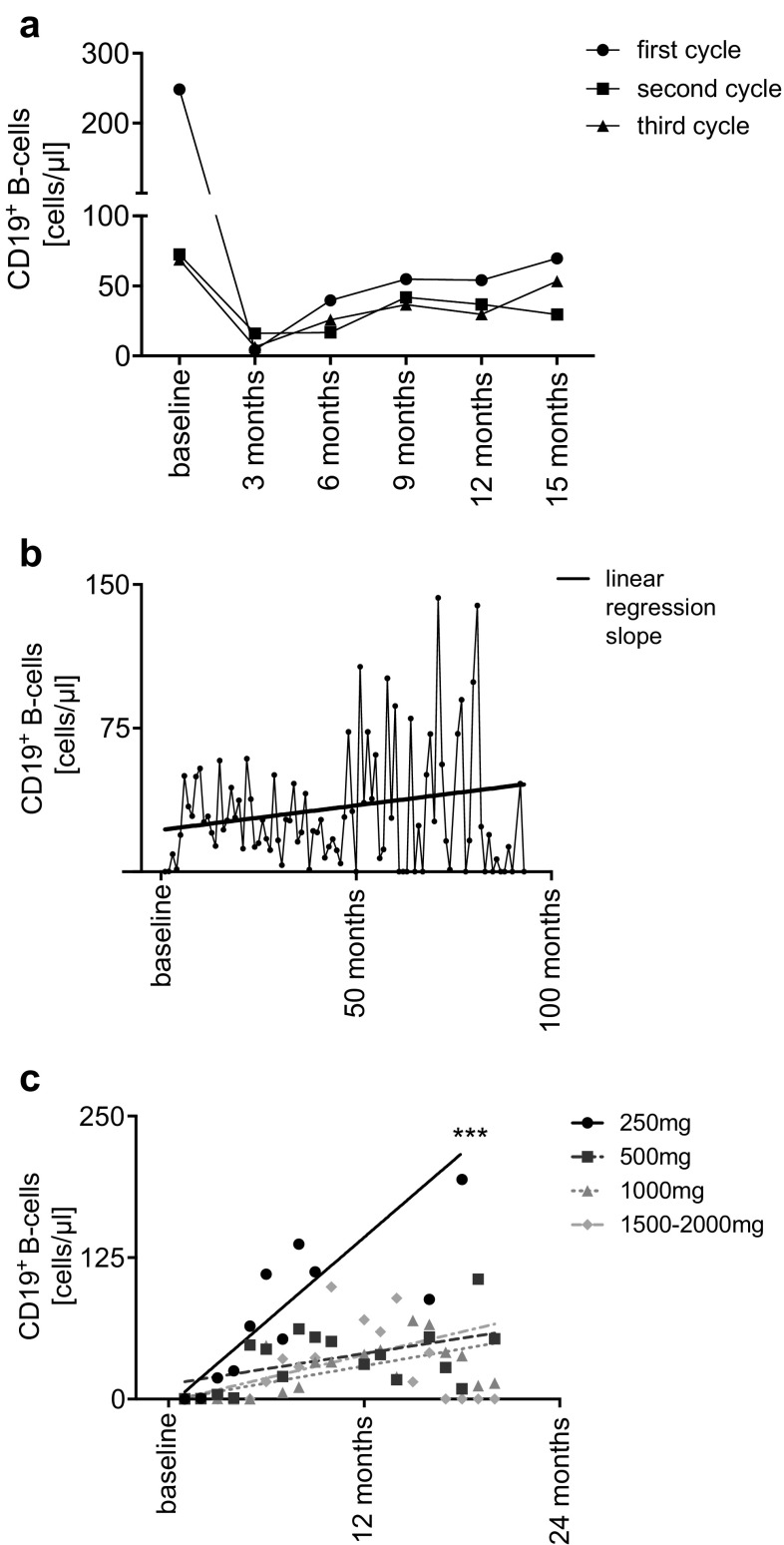
**a** Mean absolute cell count (cells/µl) of CD19^+^ B-cells over time and over multiple cycles of rituximab (RTX). Baseline represents the last available cell count before each cycle of RTX. Cell counts are summarized in 3 months intervals. **b** Mean absolute cell count (cells/µl) of CD19^+^ B-cells over time during whole follow-up after the first depletion with RTX. Linear regression slope of mean CD19^+^ B-cell counts during therapy. The regression slope slightly increases over time due to reconstitution of CD19^+^ B-cell after depletion and decreasing number of patients with redosing. For the decreasing number of patients during follow-up, the graph of mean CD19 ^+^ B-cells shows higher variance as time progresses. **c** Linear regression of absolute cell count (cells/µl) of CD19^+^ B-cells, over multiple cycles of RTX, according to applied dosage

Of 153 patients evaluated, 112 had a CD19^+^ B-cell count available at baseline (mean 248.3 ± 230.8 cells/µl). Compared to baseline there was a depletion of CD19^+^ B-cells to 4.33 ± 30.8 cells/µl (available counts *n* = 117) 3 months after first RTX application. CD19^+^ B-cells then increased to 39.66 ± 71.15 cells/µl (*n* = 58) within 6 months and to 54.91 ± 70.51 cells/µl (*n* = 39) after 9 months (Fig. 1a).

The mean number of CD19^+^ B-cells before the second RTX infusion was 72.47 ± 79.63 cells/µl (*n* = 98). Three months after the second cycle, a mean of 16.17 ± 51.18 CD19^+^ B-cells/µl (*n* = 77) was observed. Cells increased to 41.85 ± 70.55 cells/µl after 9 months (*n* = 44).

Number of CD19^+^ B-cells after the third cycle of RTX was similar to the second cycle (68.87 ± 75.08 cells/µl; *n* = 79). CD19^+^ B-cells were still largely diminished after 3 months (6.38 ± 25.85 cells/µl; *n* = 55) and the amount of CD19^+^ B-cells at the following time points did not significantly differ compared to the second therapy cycle (6 months: 25.85 ± 55.12 cells/µl; *n* = 39, 9 months: 36.65 ± 64.63 cells/µl; *n* = 29) (Fig. 1a). The course of CD19^+^ B-cells during the whole follow-up is shown in Fig. 1b.

Recovery of CD19^+^ B-cells according to dosage was analyzed by linear regression. Patients who received low dose RTX (250 mg) had a significantly faster recovery of CD19^+^ B-cells compared to 500 mg and higher doses (p*** < 0.0001; 250 mg: 12.41 ± 3.34 CD19^+^ B-cells/month, 500 mg: 2.26 ± 0.856, 1000 mg: 2.52 ± 0,455, 1500–2000 mg 3.42 ± 0.794) (Fig. 1c). Baseline characteristics of different dose groups are given in Table 3.

**Table 3:** Patients’ characteristics according to rituximab dosage

	250 mg RTX	500 mg RTX	1000 mg RTX	> 1000 mg RTX
Number of patients	23	47	36	47
Gender (female)	18	26	21	27
Mean age (years) at first RTX infusion	33 ± 10	48 ± 12	35 ± 10	40 ± 11
RRMS	14	26	17	15
SPMS	9	21	10	20
NMO/NMOSD	–	–	9	12
**Previous treatment**				
Mitoxantrone				
RRMS	1	13	6	10
SPMS	1	3	1	–
NMO/NMOSD	–	10	5	10
Natalizumab				
RRMS	6	9	5	11
SPMS	6	9	5	11
NMO/NMOSD	–	–	–	–
DMF				
RRMS	4	9	1	–
SPMS	2	2	–	–
NMO/NMOSD	2	7	1	–
Azathioprine				
RRMS	–	–	6	12
SPMS	–	–	–	–
NMO/NMOSD	–	–	6	12
Cyclophosphamide				
RRMS	–	–	1	2
SPMS	–	–	–	–
NMO/NMOSD	–	–	1	2
Glatiramer acetate				
RRMS		2	1	2
SPMS		1	1	1
NMO/NMOSD		1		1
β-Interferon				
RRMS	1	5	10	7
SPMS	1	3	3	4
NMO/NMOSD		2	7	3
Fingolimod				
RRMS	8	6	6	2
SPMS	3	4	2	1
NMO/NMOSD	5	2	4	1
Alemtuzumab				
RRMS	1	–	–	1
SPMS	1	–	–	1
NMO/NMOSD	–	–	–	–
Cladribine				
RRMS	1	–	–	–
SPMS	1	–	–	–
NMO/NMOSD	–	–	–	–
None				
RRMS	–	2	–	–
SPMS	–	2	–	–
NMO/NMOSD	–	–	–	–

All doses till 1000 mg were given as a single infusion. Doses above were given equally distributed within a 2-week interval

**Fig. 2:**
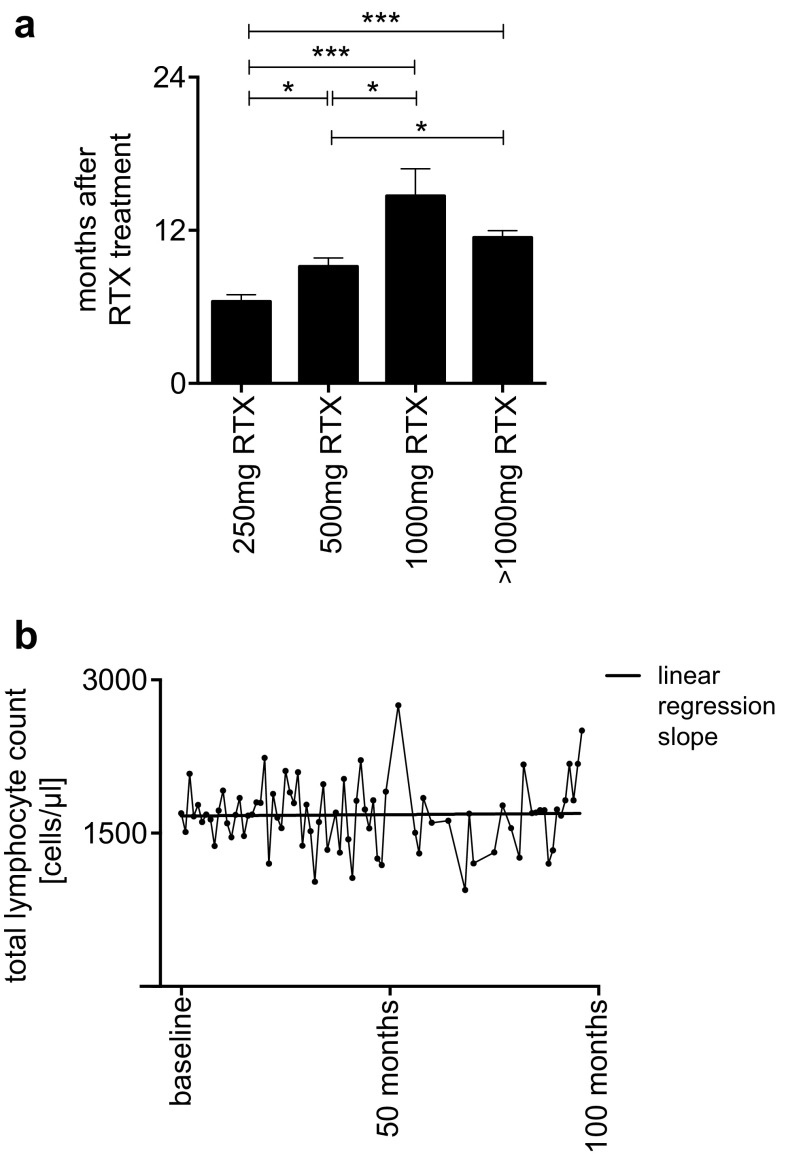
**a** Re-dosing intervals according to dosage (error bars represent SEM). **b** Mean total lymphocyte count (cells/µl) over time during whole follow-up after the first depletion with rituximab (RTX). To identify and determine the total counts of lymphocytes in the peripheral blood, flow cytometric methods were used. Linear regression slope of mean cell counts

The interval until the next infusion was significantly shorter in patients who were treated with 250 mg or 500 mg RTX (p*** < 0.001) than in patients treated with a higher dose of RTX ≥ 1000 mg (250 mg: 6.4 months ± 2.1, 500 mg: 9.1 months ± 4.4, 1500–2000 mg: 11.4 months ± 2.0). Application of 1000 mg RTX yielded the longest interval before reinfusion (mean 14.7 months ± 6.6) (Fig. 2a). B-cells at reinfusion where higher in patients treated with lower dosage but the differences were not significant (250 mg: 92.73 ± 105.20 cells/µl; 500 mg: 81.24 ± 73.99 cells/µl; 1000 mg 54.55 ± 65.92 cells/µl; 1500–2000 mg: 69.05 ± 77.24 cells/µl).

Patients diagnosed with NMO/NMOSD generally received higher dosages (1194 ± 369 mg) of RTX than patients diagnosed with MS (824 ± 824 mg; p*** < 0.0001) in shorter intervals (7.7 ± 4.3 months vs. 9.4 ± 4.2 months, *p* = 0.008) and tended to have slower recovery of B-cells per month (MS 4.02 cells/µl/months ± 2.65, NMO/NMOSD 3.69 cells/µl/months ± 5.89; *p* = 0.0685).

**Fig. 3:**
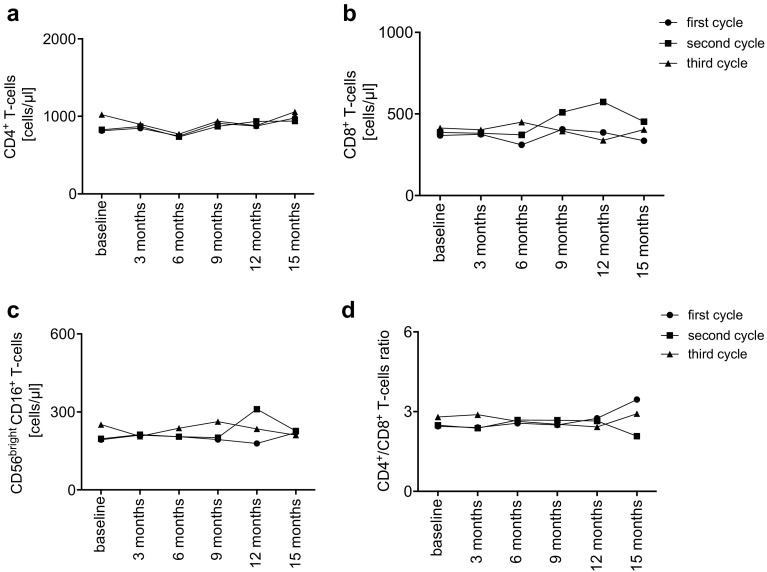
**a**–**d** Mean absolute cell count (cells/µl) of T -(CD4^+^, CD8^+^, CD4^+^/CD8^+^) and natural killer- (NK-) cells (CD56^+^CD16^+^). Baseline represents the last available cell count before each cycle of rituximab. Cell counts are summarized in 3-month intervals

Our long-term observation of total lymphocyte count over 100 months showed constant amount of cells over time (Fig. 2b). Number of CD4^+^ -cells (Fig. 3a) and CD8^+^ -cells (Fig. 3b) as well as NK-cells (Fig. 3c) only slightly varied during application interval. CD4^+^/CD8^+^ ratio did not significantly change over time (Fig. 3d).

**Fig. 4:**
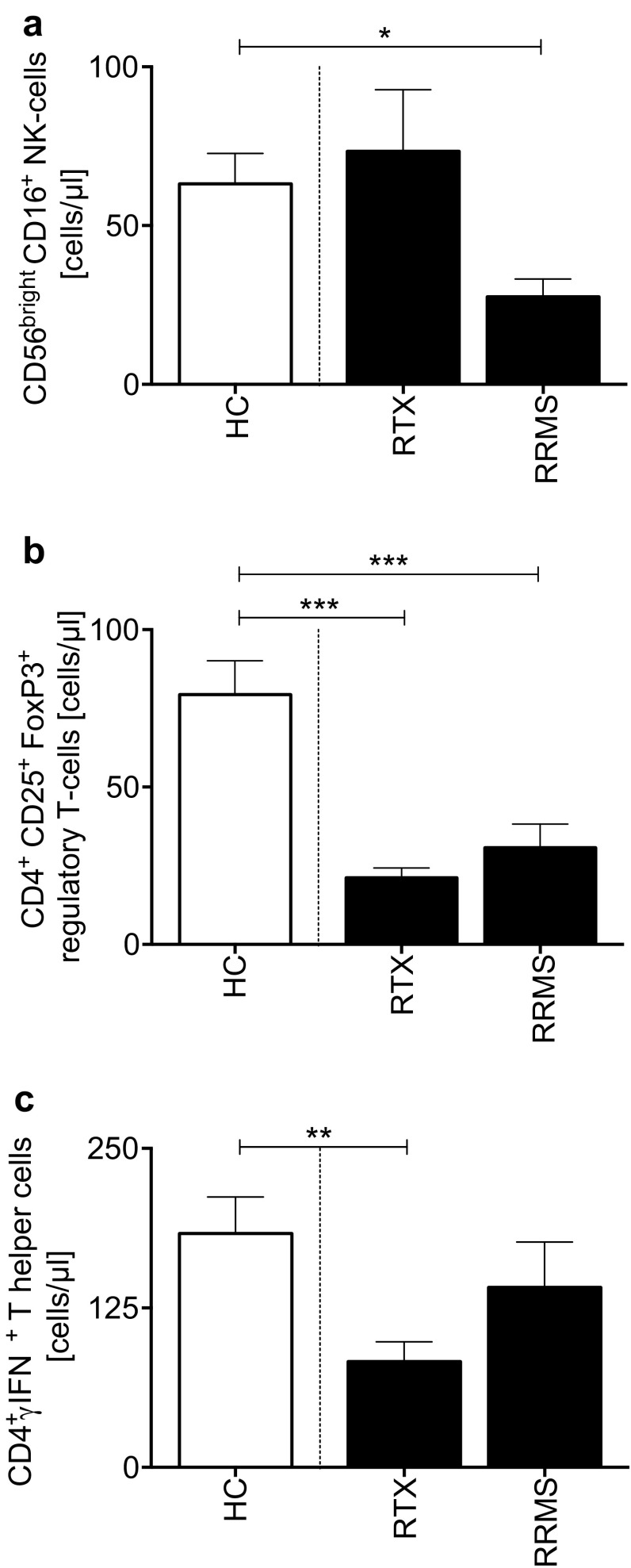
**a** Natural killer- (NK-) cells (CD56^bright^CD16^+^) and T helper -cell subsets in rituximab (RTX) treated MS vs. RRMS without RTX, and healthy controls [4]. **b** Regulatory T-cells (CD4^+^CD25^+^ FoxP3^+^), **c** Th1-cells (CD4^+^ IFNγ^+^)

In a sub-population (*n* = 25), CD56^+^ NK-, Treg- and Th1-cells were evaluated (Fig. 4a–c). NK-cells were normalized under RTX treatment and were lower in RRMS as compared with controls. Blood samples were obtained at remission. RRMS group was treated with dimethyl fumarate, detailed characteristics of the different groups are given in Table 4.

**Table 4:** Patients’ characteristics for NK- and TH1-cells

	RTX	RRMS	HC
Number of patients	25	25	25
Gender (female)	17	16	16
Age (years)	40 ± 8.7	39 ± 10	36 ± 8.2
Disease duration (years)	6.4 ± 5.9	5.3 ± 4.7	
Treatment at date of analysis	Rituximab	DMF	None
EDSS at date of analysis	4.3 ± 2.9	4.5 ± 1.9	

Data are expressed as mean ± SD where appropriate

*DMF* dimethyl fumarate, *EDSS* Expanded Disability Status Scale, *HC* healthy control, *n* number of patients, *NMO* neuromyelitis optica, *NMOSD* neuromyelitis optica spectrum disease, *RRMS* relapsing–remitting MS, *RTX* rituximab, *SPMS* secondary progressive MS

### Clinical course and MRI

Less than 40% (28/72) of patients with relapsing forms of MS and one-third (7/21) of the NMO/NMOSD patients had a relapse during the observation period. ARR in our MS cohort significantly decreased from 1.55 ± 1.36 2 years before RTX treatment to 0.26 ± 0.52 during follow-up (83% reduction, p*** < 0.0001) (Table 5). ARR in NMO/NMOSD patients decreased from 2.22 ± 1.89 to 0.42 ± 0.56 (81% reduction, p*** < 0.0001). In patients with relapse under RTX average time until first relapse after last infusion was 5.35 ± 3.04 months (Table 5). There was no correlation between B-cell counts at the time-point of reinfusion and clinical course in patients with relapses.

**Table 5:** Disease course and previous treatment

Disease course	*n* (%)	Mean ± SD
EDSS		
Baseline	153 (100)	4.5 ± 2.2
12 months	152 (96.8)	4.5 ± 2.3
24 months	101 (64.3)	4.3 ± 2.2
36 months	77 (49.0)	4.1 ± 2.2
ARR in RRMS and SPMS PE ARR in RRMS and SPMS AE	98	1.55 ± 1.36 0.26 ± 0.52
ARR in NMO/NMOSD PE ARR in NMO/NMOSD AE	21	2.22 ± 1.89 0.42 ± 0.56
Time to first relapse (observed relapses; months)	20	5.35 ± 3.05

*AE* after enrollment [ARR after enrollment was over whole follow-up period (mean 2.19 ± 1.75 years)], *ARR* annualized relapse rate, *EDSS* Expanded Disability Status Scale, *n* number of available patient data, *NMO* neuromyelitis optica *NMOSD* neuromyelitis optica spectrum disease, *PE* prior to enrollment (ARR prior to enrollment was over 2 years), *RRMS* relapsing–remitting MS, *SPMS* secondary progressive MS, *RRMS* relapsing remitting data are expressed as mean ± SD where appropriate

**Fig. 5:**
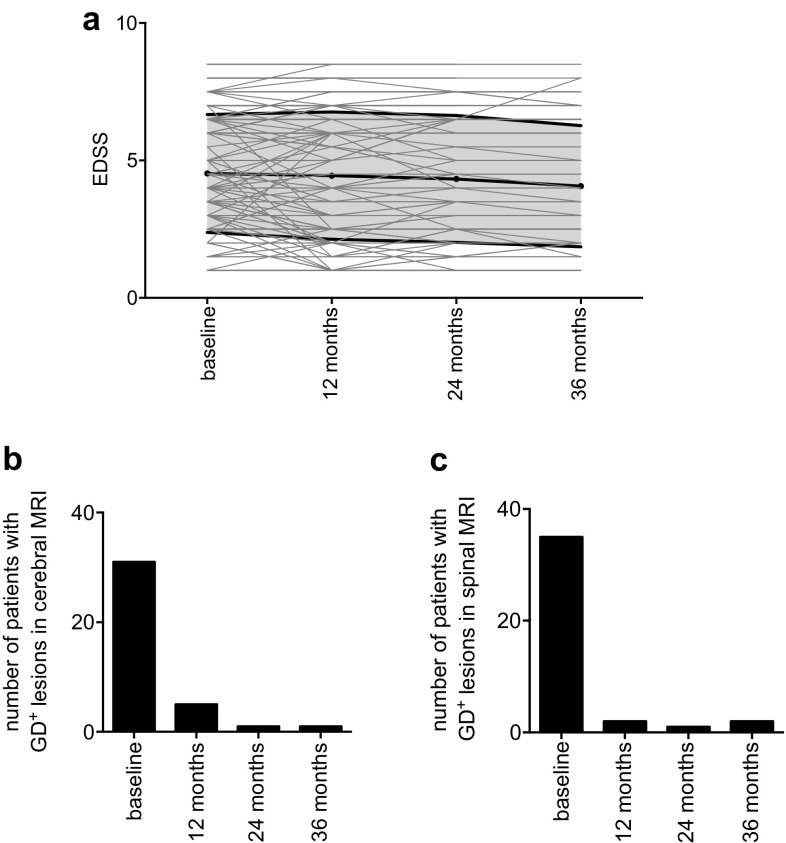
**a** Disability course as measured by EDSS over 36 months. *EDSS* Extended Disability Status Scale. Black line represents mean and SD. Lines in light gray show connecting line between individual replicated values. **b, c***Gd*^*+*^ gadolinium-enhancing lesions. Absolute number of patients with Gd^+^ T1 lesions in cerebral (**b**) and cervical spinal cord (**c**) MRI

One year after first application of RTX, 130 EDSS of the 132 MS patients were available for analyses. 32 MS patients improved, 75 remained stable, and 23 worsened (Table 6). 24/36 months after first treatment 101/77 follow-ups were available. 21/21 MS patients had a better score compared to baseline, 45/30 patients did not change and in 20/16 patients a progression of EDSS was documented (Fig. 5a; Table 6). In patients diagnosed with NMO/NMOSD 6 improved, 12 remained stable and in 3 patients a progress in EDSS was documented. At 24/36 months, 15/10 EDSS were available 4/2 improved, 6/4 remained stable and 5/4 had a higher EDSS compared to baseline.

**Table 6:** Stratification of EDSS outcome

EDSS compared to baseline	RRMS *n* (%)	SPMS *n* (%)	NMO/NMOSD *n* (%)
Available scores after 12 months	66 (p*** ≤ 0.001)	60 (p*** ≤ 0.001)	21 (p*** ≤ 0.001)
EDSS decreased	25 (38)	7 (12)	6 (29)
EDDS stable	31 (47)	42 (70)	12 (57)
EDSS increased	10 (15)	11 (18)	3 (14)
Available scores after 24 months	46 (p*** ≤ 0.001)	39 (p*** ≤ 0.001)	15 (n.s.)
EDSS decreased	17 (37)	4 (10)	4 (17)
EDDS stable	21 (46)	23 (59)	6 (40)
EDSS increased	8 (17)	12 (31)	5 (33)
Available scores after 36 months	38 (p*** ≤ 0.001)	29 (p** ≤ 0.01)	10 (n.s.)
EDSS decreased	14 (37)	7 (24)	2 (20)
EDDS stable	17 (45)	13 (45)	4 (40)
EDSS increased	7 (18)	9 (31)	4 (40)

The percentage refers to the total number of patients of one subtype at the indicated time point

*EDSS* Expanded Disability Status Scale, *n* number of patients, *NA* data not available, *NMO* neuromyelitis optica, *NMOSD* neuromyelitis optica spectrum disease, *RRMS* relapsing–remitting MS, *SPMS* secondary progressive MS

*p* value refers to number of patients with stable disease (= EDDS decreased or stable) and patients with increased EDSS according to course of MS (RRMS, SPMS) or NMO/NMOSD

Cerebral MRI scans at baseline were available for 150 patients (98%). The number of patients with Gd^+^ lesions significantly decreased during therapy (p*** < 0.001) from 31 at baseline to 5 at 12 months after treatment. Analyzing spinal MRI, number of patients with Gd^+^ lesions decreased from 35 at baseline to 2 at 12 months (p*** < 0.001). In both cerebral and spinal MRI, Gd^+^ lesions remained at low levels during the observation period (Fig. 5b; Table 7).

**Table 7:** MRI data

MRI	Total *n* = (available scans)	RRMS	SPMS	NMO/NMOSD
Cerebral T2 lesions				
Baseline	(150)	(76)	(57)	(17)
New lesions within 1st year	18 (137)	6 (68)	8 (51)	4 (18)
New lesions within 2nd year	6 (105)	3 (46)	2 (42)	1 (17)
New lesions within 3rd year	6 (74)	3 (34)	3 (29)	0 (11)
Cerebral Gd^+^ lesions				
Baseline	31 (150)	24 (76)	6 (57)	1 (17)
New Gd^+^ lesions within 1st year	5 (136)	3 (68)	2 (51)	0 (18)
New Gd^+^ lesions within 2nd year	1 (103)	0 (46)	1 (42)	0 (17)
New Gd^+^ lesions within 3rd year	1 (72)	1 (34)	0 (29)	0 (11)
Cervical T2 lesions				
Baseline	(141)	(66)	(54)	(21)
New lesions within 1st year	4 (130)	1 (62)	1 (50)	1 (18)
New lesions within 2nd year	3 (100)	2 (46)	1 (39)	0 (15)
New lesions within 3rd year	3 (74)	0 (34)	1 (29)	0 (11)
Cervical Gd^+^ lesions				
Baseline	35 (72)	16 (66)	10 (54)	9 (21)
New Gd^+^ lesions within 1st year	2 (127)	0 (62)	0 (50)	2 (18)
New Gd^+^ lesions within 2nd year	1 (99)	0 (46)	0 (39)	1 (15)
New Gd^+^ lesions within 3rd year	2 (74)	1 (34)	0 (29)	1 (11)

*Gd*^+^ gadolinium enhancing, *n* indicates the total number of patients with new lesions compared to previously available MRI. T2 and Gd^+^ lesions were counted separately, *NMO* neuromyelitis optica, *NMOSD* neuromyelitis optica spectrum disease, *RRMS* relapsing–remitting MS, *SPMS* secondary progressive MS

Due to varying infusion intervals and doses, over time clinical course was analyzed regarding mean annual RTX doses and CD19^+^ B-cell at reinfusion/relapse. We did not observe difference in mean RTX dose or CD19^+^ B-cell counts in regard of EDSS, MRI or clinical relapse (Table 8).

**Table 8:** Clinical course according to dosage/interval

	Mean annual RTX dose (mg ± SD)				CD19^+^ B-cell counts at reinfusion/relapse when applicable (cells/µl ± SD)			
	All patients	RRMS	SPMS	NMO/NMOSD	All patients	RRMS	SPMS	NMO
EDSS stable	1212 ± 793	1174 ± 787	1019 ± 770	1954 ± 838	70 ± 77	71 ± 72	91 ± 94	45 ± 81
EDSS worsened	1139 ± 702	1444 ± 1077	1071 ± 710	1822 ± 1114	67 ± 66	88 ± 86	57 ± 68	82 ± 81
*p* value	0.77	0.57	0.42	0.59	0.99	0.52	0.12	0.19
MRI stable	1257 ± 846	1260 ± 817	1009 ± 744	1784 ± 885	73 ± 79	84 ± 81	61 ± 67	56 ± 96
MRI progression	1168 ± 928	984 ± 891	983 ± 848	1842 ± 767	65 ± 77	46 ± 41	99 ± 108	57 ± 76
*p* value	0.39	0.14	0.51	0.67	0.48	0.08	0.49	0.67
Patients without clinical relapse	1284 ± 864	1148 ± 815	1022 ± 702	1727 ± 804	70 ± 79	80 ± 80	63 ± 67	47 ± 86
Patients with clinical relapse	1430 ± 947	1411 ± 924	1374 ± 1092	1986 ± 863	74 ± 80	62 ± 56	83 ± 94	75 ± 76
*p* value	0.46	0.33	0.54	0.59	0.76	0.46	0.96	0.27

Mean annual RTX dose = mean dose applied between the recorded variables (EDSS, MRI) during whole follow-up. MRI progression designates all new gadolinium-enhancing or new T2 lesions compared to previous MRI (cerebral and spinal) during whole follow-up

For patients with a relapsed CD19^+^ B-cell count indicates the first analysis/cell count after relapse and before re-dosing. For patients without a relapse, it is defined as highest available cell count before re-dosing

Data are expressed as mean ± SD where appropriate

*EDSS* Expanded Disability Status Scale, *NMO* neuromyelitis optica, *NMOSD* neuromyelitis optica spectrum disease, *RTX* rituximab, *RRMS* relapsing–remitting MS, *SPMS* secondary progressive MS

### Side effects

Only major side effects were recorded. One patient was hospitalized for severe pneumonia. In one patient, a reactivation of hepatitis B was observed. In general, there were rare side effects and RTX was well tolerated.

## Discussion

There is growing evidence for the efficacy of B-cell depleting therapies in various autoimmune diseases [4]. This has previously resulted in the FDA approval of RTX for the treatment of rheumatoid arthritis in 2006. Two phase II trials in MS, and several open label trials in MS and NMO/NMOSD underlined the efficacy also in MS and NMO/NMOSD with a favorable safety profile of the anti-CD20 monoclonal antibody RTX. Finally, its successor, the humanized antibody ocrelizumab (Ocrevus^®^, Roche, Switzerland) was recently approved for the treatment of RRMS and PPMS by the FDA and the EMA.

Yet, the RTX dosages used significantly vary in different cohorts, and ocrelizumab is approved only at a defined dosage. Hence, in our study, we evaluated the long-term depletion and repopulation rate of peripheral CD19^+^ B-cells under RTX treatment as a potential surrogate marker for the clinical outcome.

We showed that CD19^+^ B-cell repopulation was significantly faster when 250 mg RTX was applied. Higher doses of RTX (500–2000 mg) did not lead to sustained B-cell depletion, which might indicate a ceiling effect. Second, long-term RTX treatment did not induce a substantial change in total T-cell populations over time. In a long-term manner, the number of CD4^+^ - and CD8^+^ -cells, as well as CD4/CD8 ratio did not change significantly compared to pre-RTX levels. This is well in line with previous studies [13]. We did not observe any severe side effects such as secondary autoimmunity (SAI). This is in contrast to T- and B-cell-depleting therapies, i.e. alemtuzumab, where SAI are seen in more than 30%. These side effects are attributed to an excessive repopulation of B cells accompanied by the depletion of (regulatory) T-cells [1, 5].

Moreover, reduced numbers of CD19^+^ B-cells were associated with reduced ARR, EDSS and reduced numbers of Gd^+^ enhancing lesions. Besides the small number and different treatment regimens, NMO/NMOSD and MS patients did neither show significant differences in intensity, nor duration of cell depletion.

Continuous monitoring of CD19^+^ B-cells may be a sufficient tool for an individualized decision making on dosage and reinfusion intervals of B-cell depleting therapies. Fixed dosage and infusion intervals, as in therapy regime of ocrelizumab, may explain the increased risk for infections and malignancies. In our cohort, dosage of RTX ranging 500–1000 mg led to longer reinfusion intervals. Higher dosages of RTX neither lead to extended reapplication intervals, nor any additional treatment effects, and may be avoided [16].

Despite the therapeutic effect being closely associated with the absence of CD19^+^ B-cells, we did not observe a correlation between B-cell counts at the time-point of reinfusion and clinical course or MRI outcome in patients in whom relapses did occur. This might be due to different proportion of progenitor CD19^+^ B-cells and mature B-cells after the first cycle of RTX treatment. After replenishment of the B-cell compartment there are mainly naïve B-cells following repletion of circulating B-cells. CD27^+^ memory phenotype cells stay at significantly lower levels in peripheral blood [13]. It has been recently shown that memory B cells drive proliferation of self-reactive brain-homing CD4^+^ T-cells in patients with multiple sclerosis and that RTX strongly reduces autoproliferation and proinflammatory cytokine responses [10]. This may be one of the reasons for the long-lasting beneficial clinical effects of anti-CD20 therapy [13, 15]. However, B-cell responses in MS patients have shown to be per se altered in MS [3]. We observed an increase of B cells over time that is due to statistical effects and patients’ behavior. Number of patients being analyzed decreased over time and simultaneously variations in CD19^+^ B-cell counts increased as there were single statistical outliers.

Our study has several limitations, the main being its retrospective character. Another limitation arises from the different dosages used over time. The varying infusion protocols reflect the evolution of RTX titration toward lower dosage over the past 10 years in off-label use, and allow the differentiated analysis of B-cell repopulation rates.

Nevertheless, we could build up a robust database over a long-term period of 100 months with a sufficiently large number of patients. Theoretically, other immune cell subsets bearing CD20 antigen may contribute to the efficacy of RTX and may be overlooked in FACS analysis-guided infusion cycles. At least, in our cohort, the clinical effect was very robust.

In summary, in contrast to treatment of rheumatoid arthritis or lymphoma, 500 mg or even 250 mg RTX in intervals of approximately 6–9 months are an effective and at the same time safe therapy option in MS. In our cohort, higher dosage in elongated intervals proved equally effective. However, dosages beyond 1000 mg did not reveal additional effects in regard to B-cell depletion, administration interval and clinical outcome. Given the large inter-individual range of B-cell recovery time, we suggest that the CD19^+^ B-cell repopulation rate may serve as surrogate marker to appraise individually adapted therapy intervals. Although the next generation of anti-CD20 monoclonal antibodies is approved for the treatment of MS, many mechanistic aspects and long-term immunological consequences remain unclear. Analysis of more than 10 years of RTX use in MS may shed further light into these questions.
